# HIV self‐testing in India: implementation and qualitative evaluation of a web‐based programme with virtual counsellor support

**DOI:** 10.1002/jia2.26302

**Published:** 2024-06-11

**Authors:** Rose Pollard Kaptchuk, Jalpa Thakker, Jade Bell, Saya Okram, Usha Gopinath, Shruti H. Mehta, Ajay Kumar Reddy, Talia A. Loeb, Visvanathan Arumugam, Samit Tandon, Mugundu Ramien Parthasarathy, Subash Chandra Ghosh, Aditya Singh, Deepika Srivastava Joshi, Sukhvinder Kaur, Sunil Suhas Solomon, Allison M. McFall

**Affiliations:** ^1^ Department of International Health The Johns Hopkins Bloomberg School of Public Health Baltimore Maryland USA; ^2^ Division of Infectious Diseases Department of Medicine Johns Hopkins School of Medicine Baltimore Maryland USA; ^3^ Y.R. Gaitonde Centre for AIDS Research and Education Chennai India; ^4^ Department of Epidemiology The Johns Hopkins Bloomberg School of Public Health Baltimore Maryland USA; ^5^ Blue Lotus Advisory New Delhi India; ^6^ United States Agency for International Development Washington DC USA

**Keywords:** HIV care continuum, self‐testing, India, diagnostics, key and vulnerable populations, linkage to care

## Abstract

**Introduction:**

To achieve epidemic control of infectious diseases, engaging higher‐burden populations with accessible diagnostic services is critical. HIV self‐testing (HIVST) is a promising option.

**Methods:**

We implemented an online HIVST programme for key populations across India. Eligible clients were 18 years or older, self‐reported a negative or unknown HIV status and reported not taking antiretroviral therapy. Clients who reported a prior HIV diagnosis were not eligible to receive an HIVST kit. HIVST clients received kits via courier or in person at pre‐determined pick‐up points supported by trained counselling staff. Virtual counsellors engaged clients online and by phone and offered support to register, access, and complete HIVST free of cost. Virtual counsellors supported clients to report results and engage with follow‐up services. Follow‐up included linking clients with a positive result to confirmatory testing and HIV care services. We assessed programmatic data across HIV continuum outcomes and conducted a qualitative evaluation through interviews with purposively sampled clients.

**Results:**

Between 30 June 2021 and 30 September 2022, 5324 clients ordered an HIVST kit (76% men, 13% women, 7% transgender people, 4% unknown gender). Of the 4282 clients reporting results (94% of those who received a kit), 6% screened positive, among whom 72% (*n* = 184) completed confirmatory testing. Themes from 41 client interviews included satisfaction about the convenience and privacy of services and the discreet nature of kit delivery. Respondents were drawn to the convenience of HIVST and appreciated gaining courage and comfort throughout the process from virtual counsellor support. For respondents who screened positive, challenges to care linkage included fearing judgemental questions from public providers and wanting more time before starting treatment. Clients shared concerns about kit accuracy and suggested that instructional materials be provided with more diverse language options.

**Conclusions:**

Web‐based HIVST services with tailored support appeared to facilitate HIV service access and engagement of harder‐to‐reach populations across India. Assistance from a community‐oriented counsellor proved important to overcome literacy barriers and mistrust  in order to support the HIVST process and service linkage. Learnings can inform global efforts to improve the critical step of diagnosis in achieving epidemic control for HIV and other infectious diseases.

## 1 INTRODUCTION

Globally, infectious disease programmes have ambitious targets to achieve epidemic control or elimination. UNAIDS has established 95‐95‐95 targets for HIV wherein 95% of people living with HIV (PLHIV) are aware of their status, 95% of those aware are retained on antiretroviral therapy treatment and 95% of those on treatment achieve viral suppression by 2030 [1]. Diagnosis plays a critical role in achieving targets. Diagnosis, especially among harder‐to‐reach populations, was already challenging prior to the COVID‐19 pandemic. However, since the pandemic, diagnosis of new HIV acquisitions dropped by nearly 50% in sub‐Saharan Africa [2]. In India, similar trends at HIV testing centres show a decrease of about 50% in new HIV diagnoses following the pandemic [3, 4, 5]. Innovative strategies to improve diagnoses in this “new normal” are essential not only for achieving infectious disease programme goals, but also for expanding equitable access to testing services.

HIV self‐testing (HIVST) is a strategy where people screen themselves for HIV acquisition either with or without supervision. While HIVST has been shown to improve HIV screening among general and key populations (populations facing higher risk for HIV acquisition such as men who have sex with men and transgender people) [6], programmes have also noted drop‐offs between initial screening for HIV and treatment initiation, especially in field‐based testing programmes [7]. Additional data are needed to guide programmes on how best to implement HIVST and ensure engagement along the entire continuum of care, particularly given HIVST's potential for cost savings [8, 9].

India, with 2.5 million PLHIV, is home to the second largest population of PLHIV globally [10]. Based on estimates from 2022 to 2023, 79% of PLHIV in India are aware of their HIV status. Among them, 86% are on antiretroviral therapy (ART), and among those tested for HIV viral load, 93% are virally suppressed [10]. This progress highlights the need to expand access to HIV testing to accelerate progress towards achieving the first UNAIDS 95‐95‐95 target. In contrast to other countries with large numbers of PLHIV, the general population prevalence in India is 0.21% [10], with the epidemic largely concentrated among key populations. The Government of India estimates a higher HIV burden among people who inject drugs (9.03% prevalence), transgender people (3.78% prevalence), men who have sex with men (3.26% prevalence) and female sex workers (1.85% prevalence) [10]. A 2022 survey in Pune, India showed that 78% of transgender persons living with HIV were aware of their status [11]. A 2022 study across 21 cities in India estimated that 78% of men who have sex with men in India living with HIV and 49% of people who inject drugs living with HIV are aware of their status, demonstrating a similar need among key populations to improve access to HIV testing [12]. The penetration of high‐speed internet access and online dating platforms in India (e.g. Grindr, Blued, Planet Romeo, etc.) have resulted in many key population members seeking partners online; however, these virtual populations are harder to reach by routine HIV programming. Accordingly, we launched an online platform incorporating HIVST to improve access to health education and HIV screening services to virtual populations in India.

We present 1 year of programmatic data (2021−2022) and a qualitative evaluation among HIVST programme clients. Our assessment aims to inform global efforts to improve diagnosis and linkage to care services for HIV and other infectious diseases as online engagement expands.

## 2 METHODS

### 2.1 Service setting

We designed a web‐based platform, called Safe Zindagi, as a hub for HIV continuum of care education and service linkage resources serving Indian clients supported by the US Agency for International Development/US President's Emergency Plan for AIDS Relief (USAID/PEPFAR). Informed by WHO guidelines on HIVST, we launched HIVST services as part of Safe Zindagi in June 2021. Our platform distributed the MORCHECK 3rd generation oral mucosal transudate rapid HIVST kit, which was approved by the Drug Controller General of India with the condition that they included instructions for use. Kits were made available free of cost to eligible clients.

### 2.2 Population and recruitment

Resources and services on Safe Zindagi were not restricted to any population or geography. Online demand generation included promotional posts on Safe Zindagi social media handles and paid advertisements on social media sites including Facebook and Instagram. Virtual counsellors (VCs) trained in sexual health management also conducted direct outreach through social media platforms, WhatsApp and online dating applications aiming to reach sexual minority individuals. VCs created social media profiles with bios containing information about Safe Zindagi. They were trained to chat with clients with standardized messaging about sexual risk, HIV and the services offered on our virtual platform. They engaged directly with clients online and chatted through social media sites and WhatsApp to navigate all people interested to our virtual platform. By sending personalized links, a client was assigned the same VC who reached out to them online for HIVST services. Offline demand generation included promotion among private providers and community‐based organizations already engaging with key populations. We also worked with government testing/treatment centres and community‐based screening sites to offer HIVST through Safe Zindagi as an option for partner testing services (index testing) targeted at partners who were unwilling to visit a facility for testing. To register for HIVST services, clients had to report being 18 years old or older, HIV negative or of unknown status and not taking treatment to manage HIV.

### 2.3 HIVST service process

**Figure 1:**
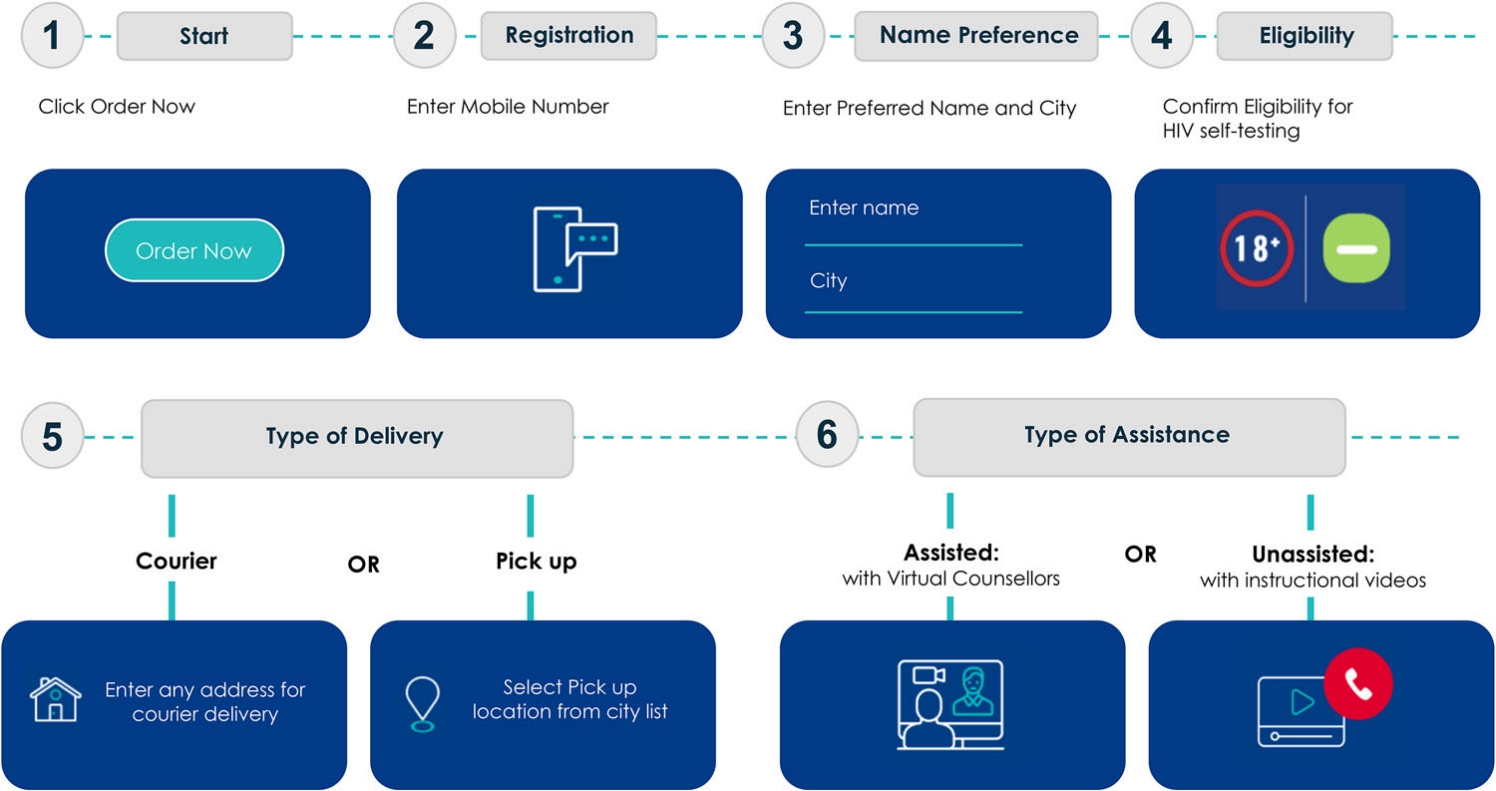
Web‐based HIV self‐testing client procedures and flow.

Demand generation activities directed clients to the Safe Zindagi website to register and order a free HIVST kit which could either be delivered to any address or picked‐up at a pre‐determined location. Before initiating services, clients were provided information about HIV as well as public HIV services in India and gave their consent to be contacted by a VC. After registering on the Safe Zindagi platform, every client was assigned a VC. Clients had the option to change their assigned VC at any point. Previous HIVST clients could request a repeat test after 3 months (or earlier if approved by a VC). Clients selected their preferred mode of HIVST delivery (mail courier to any address in India or pick‐up at specified locations—called pick‐up points [PUPs]) and whether they wanted assistance from a VC when completing HIVST (Figure 1). We developed multimedia communication materials in Hindi, Telugu and English. These included a pamphlet inserted into HIVST kit boxes with step‐by‐step instructions on using the kit, and instructional animated videos with voiceover which VCs sent to clients registered through Safe Zindagi. All informational materials were pretested among community representatives from Maharashtra, Telangana and Delhi. We gathered and incorporated community feedback prior to finalizing informational materials.

Clients were instructed to upload a picture of their HIVST result to the web platform along with the kit's barcode to ensure the kit belonged to the client. In addition to automated notifications sent through SMS/WhatsApp about completing HIVST steps, VCs remained in contact with clients via SMS/WhatsApp to conduct pre‐test counselling, support HIVST completion, provide post‐test counselling and help upload results. Those with a positive HIVST result received location options for confirmatory testing and treatment centres. VCs remained in contact with screened‐positive clients to support service linkage and treatment adherence. Communication frequency depended on client preference. VCs could register and upload pictures/documents on behalf of a client. VCs received a standard salary with additional payment based on the achievement of successful client linkage across HIVST continuum outcomes.

Clients could also receive HIVST directly from PUPs without prior registration (considered in programme data as a type of PUP order called “walk‐in”). PUPs were established in USAID‐determined implementation districts across Maharashtra and Telangana states in accessible locations for clients, including private clinics, pharmacies and community‐based organizations. PUPs had kits onsite, a safe testing space for clients, and staff trained on the online platform and HIVST. We identified a staff member with counselling background and experience working with key population communities at each PUP to provide counselling to HIVST clients. PUP staff helped register HIVST clients through the Safe Zindagi platform, supported HIVST completion and result upload, and provided pre‐ and post‐test counselling and linkage. The process of registration on the platform, including being assigned a VC, remained the same for walk‐in clients. Following their experience at the PUP, VCs followed up with walk‐in clients to support service linkage.

### 2.4 HIVST programme data and analyses

We analysed programmatic data from 30 June 2021 to 30 September 2022 to describe client characteristics and HIVST continuum outcomes. HIVST continuum outcomes measured were: (1) ordering an HIVST kit; (2) receiving the kit; (3) uploading the testing result; (4) receiving a confirmatory test (if HIVST positive); and (5) treatment initiation (if confirmed HIV positive). Information across all steps was entered into the Safe Zindagi platform either by clients themselves, VCs or PUP staff. Inventory managers and PUP staff scanned barcodes of HIV self‐test kits which documented every kit dispatched to a client. For clients who screened positive and completed confirmatory testing, VCs uploaded test results received either directly from laboratories or from clients. Due to repeat orders, outcomes are presented in terms of orders placed as well as clients reached. We also examined order type (PUP, courier, walk‐in) and whether or not assistance was given during the completion of HIVST. Additionally, percent positivity from both HIVST and confirmatory testing results was calculated. To reduce barriers to ordering, questions on demographics and risk behaviours were asked but not required, resulting in a high proportion of missing data.

### 2.5 Qualitative programme evaluation

We conducted a qualitative evaluation of the HIVST programme. Qualitative in‐depth interviews with respondents who ordered an HIVST kit from the platform were conducted over the phone in Hindi or English from December 2021 to July 2022. Respondents were selected from a list of clients who had previously agreed to be contacted later about their experiences and sampled for representation across geographic region, gender, age, kit retrieval method and HIVST result. Interviews were conducted by an Indian interviewer experienced in qualitative methods who was not involved with service delivery. The interviewer contacted clients directly for the interview. Some clients were recruited with help from their VC, who facilitated initial contact. The interviewer conducted oral consent with clients over the phone prior to starting interviews. Based on a purposeful balance of these attributes, we attempted to contact 342 clients for an interview and successfully reached 244. Among them, 25 did not speak Hindi or English and 178 were either unavailable or not interested to complete an interview. We conducted 41 interviews with consenting respondents (17% of those we successfully contacted). Interviews were audio recorded, except for clients who did not give consent to record the interview, in which case the interviewer took detailed notes. The pre‐tested interview guide included questions about placing HIVST orders, retrieving the kit, completing HIVST and interpretating results, interaction with VCs and suggestions to improve services. Interviews were conducted in Hindi and/or English. Respondents were compensated INR 300.

Audio recordings of interviews were transcribed and translated to English by the interviewer. Three analysts, including the interviewer, collaboratively developed a codebook using an inductive thematic approach. Analysts independently applied codes to one transcript and discussed differences in coding choices. Discussion synthesized understandings of codes and themes to refine the codebook. One analyst then applied codes to the remaining interviews. The three analysts discussed coded content across themes and compared findings across respondent characteristics. Coding and analysis were done using Dedoose Version 9.0.54.

The Johns Hopkins Bloomberg School of Public Health Institutional Review Board reviewed and approved these activities as public health surveillance and thus not human subjects research. Interview respondents were provided a description of the assessment activity and an opportunity to ask question and gave oral consent before participation. The funders of the HIVST programme and evaluation (USAID and the PEPFAR) received regular updates about implementation design, data collection and data analysis. Data collection and interpretation were carried out independently by the authors. HIVST kits were donated by Invex Health Private Limited.

## 3 RESULTS

### 3.1 HIVST programme outcomes

**Figure 2:**
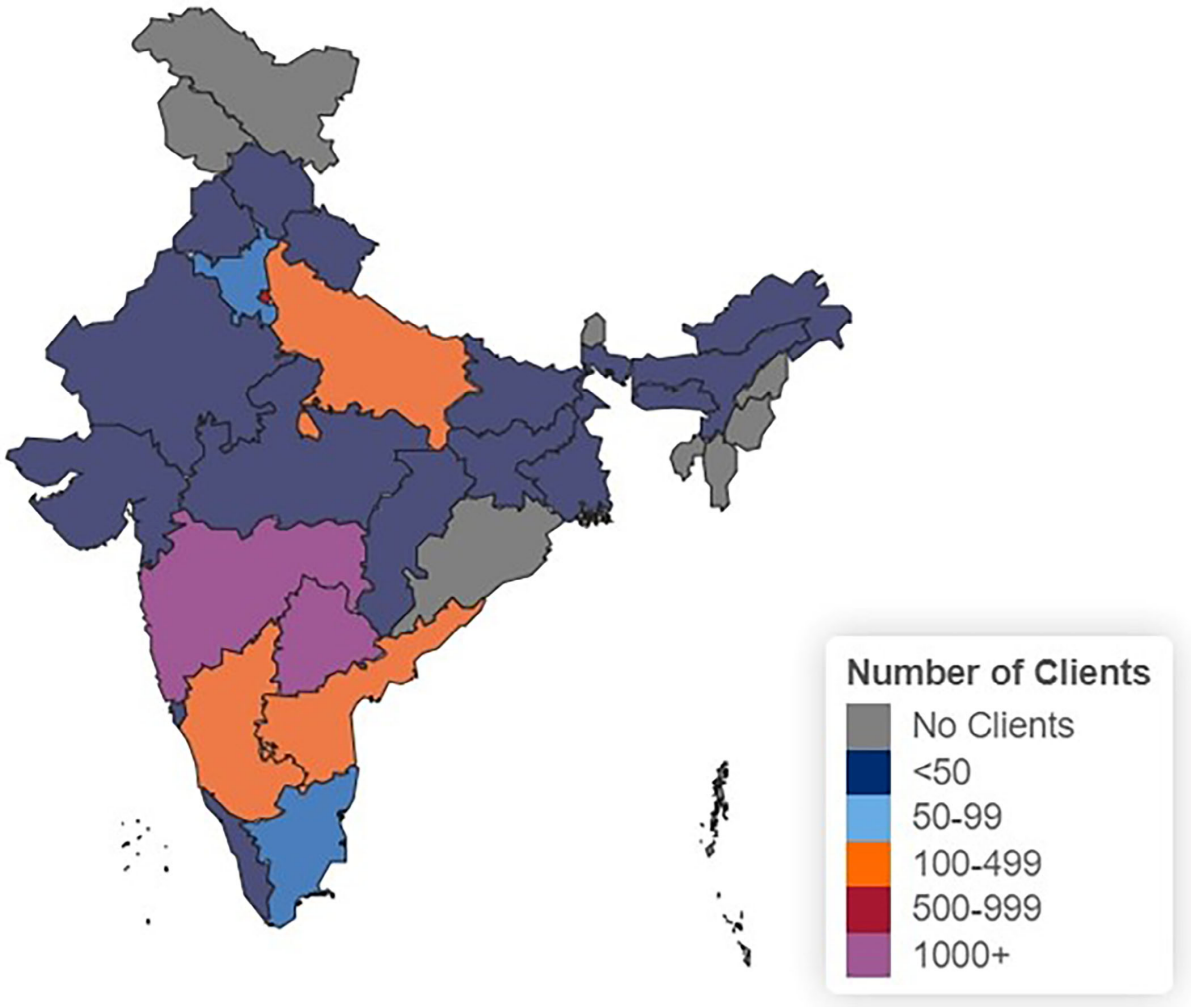
Distribution of HIV self‐testing clients by Indian state and union territory.

Between 30 June 2021 and 30 September 2022, a total of 5324 clients registered and ordered an HIVST kit. The median age of clients was 26 years (interquartile range 24−38) and 74% identified as men (Table 1). Of the 35% with available data about sexual behaviours, 79% identified as men who have sex with men. Clients from the southern and western regions of India accounted for 68% of those who have ordered an HIVST kit. With in‐person PUPs offered only in Telangana and Maharashtra, these states made up 20% and 37% of clients, respectively (Figure 2). Of the 3597 clients who responded about prior testing, 1897 (53%) said they had never tested for HIV before.

**Table 1:** Demographics and HIV risk among HIV self‐testing clients, overall and by result

*n* (%)/median (interquartile range)	All clients	Kit not received or no result reported	HIV self‐testing screened negative	HIV self‐testing screened positive
*N*	5324	1042	4028	254
Median age (interquartile range)	26 (23−30)	26 (23−30)	26 (23−31)	27 (24−38)
Gender				
Man	3915 (74%)	797 (76%)	2926 (73%)	192 (76%)
Woman	670 (13%)	99 (10%)	538 (13%)	33 (13%)
Transgender	367 (7%)	27 (3%)	321 (8%)	19 (7%)
Unknown	372 (7%)	119 (11%)	243 (6%)	10 (4%)
Region				
North	655 (12%)	144 (14%)	492 (12%)	19 (7%)
Central	512 (10%)	101 (10%)	394 (10%)	17 (7%)
East & Northeast	73 (1%)	29 (3%)	42 (1%)	2 (1%)
West	1997 (38%)	182 (17%)	1720 (43%)	95 (37%)
South	1620 (30%)	469 (45%)	1042 (26%)	109 (43%)
Unknown	467 (9%)	117 (11%)	338 (8%)	12 (5%)
Registration type				
Self	2607 (49%)	848 (81%)	1683 (42%)	76 (30%)
Virtual counsellor	430 (8%)	67 (6%)	345 (9%)	18 (7%)
Pick‐up point	2287 (43%)	127 (12%)	2000 (50%)	160 (63%)
Self‐identified key population group				
People who inject drugs	26 (0.005%)	1 (%)	25 (1%)	0
Men who have sex with men	1501 (28%)	180 (17%)	1248 (31%)	73 (29%)
Transgender people	212 (4%)	13 (1%)	184 (5%)	15 (6%)
Female sex workers	150 (3%)	5 (%)	139 (3%)	6 (2%)
Unknown	3435 (65%)	843 (81%)	2432 (60%)	160 (63%)
Risk of HIV^a^				
High	1867 (35%)	224 (21%)	1525 (38%)	119 (47%)
Medium	1089 (20%)	166 (16%)	880 (22%)	42 (17%)
Low	323 (6%)	80 (8%)	231 (6%)	12 (5%)
None	54 (1%)	23 (2%)	31 (1%)	
Unknown	1991 (37%)	549 (53%)	1361 (34%)	82 (32%)
Number of orders				
One order	4875 (92%)	1018 (98%)	3614 (90%)	243 (96%)
Multiple orders	399 (7%)	24 (2%)	414 (10%)	11 (4%)

^a^ Based on self‐reported responses to an optional risk behaviour assessment which asked about sexual partners, condom use, transactional sex, substance use, pre‐exposure prophylaxis use, forced sex and sexually transmitted infection diagnoses in the past 6 months.

### 3.2 HIVST kits ordered

**Figure 3:**
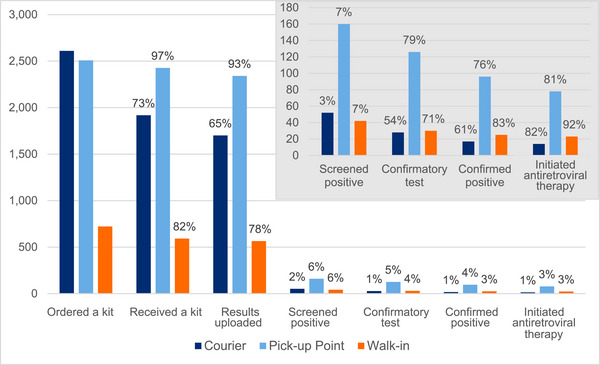
HIV self‐testing cascade outcomes by order type. *Note*: The main graph shows the percentage at each step in the process out of all HIV self‐testing kit orders. The subset graph shows the percentage out of the previous step in the cascade.

The total number of kits ordered was 5840. This includes kits for previous clients who ordered another test in the future, called repeat orders. Seven percent of clients had repeat orders. Of all kit orders, 85% (*n* = 4937) kits were received, and 93% (*n* = 4607) tests were completed with results uploaded. About half of the kits which did not reach clients were due to the client cancelling the order; other reasons were an incomplete address or incorrect address. Among results uploaded, 74% completed the kit with assistance from the VC. Among all orders, 45% were PUP orders, 43% were couriered and 12% were walk‐ins. Figure 3 depicts each step of the HIVST process by order type of each kit ordered. We observed a higher positivity from the HIVST kit among PUP and walk‐in orders compared to courier orders.

### 3.3 Clients reached

**Figure 4:**
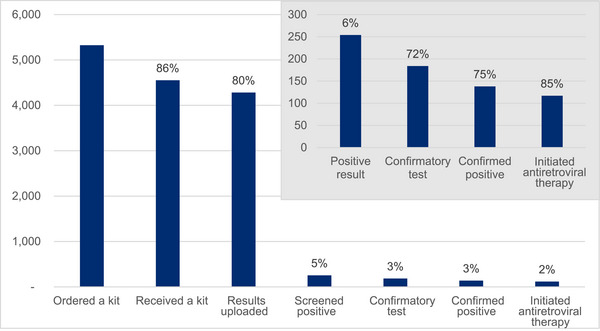
HIV self‐testing cascade outcomes by client. *Note*: The main graph shows the percentage at each step in the process out of all HIV self‐testing clients. The subset graph shows the percentage out of the previous step in the cascade.

Of the 5324 unique clients who ordered a kit, 86% (*n* = 4553) received a kit, among whom 94% (*n* = 4282) completed the test and uploaded the result (Figure 4). Of those, 6% (*n* = 254) screened positive, among whom 72% (*n* = 184) completed confirmatory testing. Seventy‐five percent (*n* = 138) were confirmed positive, among whom 85% (*n* = 117) were successfully linked to ART. The majority of confirmed positive clients were those from PUP orders (70%), with courier and walk‐in orders contributing 12% and 18%, respectively.

### 3.4 Qualitative evaluation results

We completed 41 interviews with HIVST clients (29 men, 8 women, 4 transgender clients). Respondents varied in age from 19 to 45 years old, with over half (54%) between 20 and 29 years old. Most respondents completed HIVST registration online either by themselves (44%) or via a VC (39%). The remaining completed HIVST registration at a PUP (17%). Of those interviewed, seven screened positive through HIVST.

#### 3.4.1 Interest in HIVST

Respondents shared reasons why they were initially interested in HIVST. These included the convenience of free services, doing HIVST anywhere and receiving results quickly in order to have the assurance from learning their HIV status (Table 2).

**Table 2:** HIV self‐testing client quotes from qualitative assessment

Theme	Client quote
Interest in HIV Self‐testing: Assurance of HIV status	*“We do not have time to think of ourselves and health. Sometimes we are engaged in risky behaviour but we ignore it…at least with this self‐test kit, we can clarify our doubts.” (​Transgender participant, age 28)​*
HIV Self‐testing Programme Logistics: literacy and privacy	*“If a person is illiterate, they cannot use the website at all. People in our [transgender] community, many are illiterate and they also usually do not have trust in other people. They are scared of many things in the society…they will have doubts on sharing this information and they have problem with trust. For me, I knew him [the virtual counsellor]. So, I could trust him.” (Man participant, identified as bisexual and part of transgender community, age 40)*
HIV Self‐testing Receipt: courier service	*“HIV is still stigmatized in our society. If my parents found out that I was doing an HIV test, obviously they would ask why. So I thought it would be better if I myself received the package at the office.” (Man participant, age 26)*.
Pre‐test Planning: testing alone	*“​​You know, [HIV testing] is a very sensitive issue. I do not want to show to other people that I am doing an HIV test and I am homosexual. If I go to a clinic for a test, people who are working there or people nearby the clinic might see me entering the clinic. I might feel uncomfortable and guilty. But here [referring to self‐testing], nobody knows what is in this package. It is very much in privacy.” (Man participant, age 45)*
Completing HIV self‐testing: Virtual Counsellor support	*“Talking to the counsellor helps in gaining trust with the service and it definitely gives us the confidence of using and doing the test…I would say talking to the counsellor was really helpful. It helps in building trust in this website too.” (Man participant, age 19)*
Experience with Virtual Counsellor: emotional support	*“[VC name] told me before that I do not need to be worried even if the result is positive…for the mental support he called me several times. He was telling me again and again ‘don't worry whatever the result is’…I was very relaxed and relieved.” (Man participant, age 26)*
Screening Positive: starting treatment	*“I am not taking any medicine now. I know it is necessary to take medicine…[but] I need to think about all this. I am also worried that my family might know that I have this [HIV]. So, I told the hospital staff and my partner, I need time.” (Man participant, age 32)*

#### 3.4.2 HIVST programme logistics

Respondents shared satisfaction with the logistics of the HIVST service. Clients said it was easy to navigate the website to register and order an HIVST kit and appreciated that the instructions were in both English and Hindi. Participants said that they understood the instructions on the pamphlet included with the kit and instructional videos but suggested that instructional material should be offered with more diverse language options. When participants had questions about the process, they relied on friends or their VC to clarify. Respondents spoke about feeling apprehension to share personal information during registration, and how talking with their VC helped assuage doubts. One respondent discussed how literacy barriers and privacy concerns converge (Table 2).

#### 3.4.3 HIVST receipt

Privacy also came up when respondents talked about receiving HIVST kits, often in regard to avoiding judgement. For courier deliveries, clients valued discreet packaging which did not reveal its contents. This provided a sense of autonomy and relief to receive a kit without having to justify it to a family member or someone they lived with. Another strategy to preserve privacy was to avoid home deliveries entirely (Table 2). Non‐home delivery locations included a work office, private space near home or a store.

#### 3.4.4 Pre‐test planning

Respondents talked about how pre‐planning was essential to ensure a comfortable HIVST experience. Two participants chose to do HIVST with someone else, either a close friend or relative, to provide immediate access to moral and social support. Others feared facing unnecessary questions or judgement and prioritized being alone when self‐testing (Table 2).

#### 3.4.5 Completing HIVST

Respondents shared concerns and anxiety they experienced throughout the process, including scepticism about the accuracy of the test kit results. Respondents felt nervous and restless waiting for the kit result, troubled by the idea that results could be invalid or show false results. They reported feeling nervous about testing positive and dealing with the consequences. VCs spoke with clients via voice calls and WhatsApp messages before and during the process of HIVST which helped to answer questions and provide mental strength and courage. Respondents described VCs as professional, easy to talk to, helpful and comforting (Table 2).

#### 3.4.6 Experience with VCs

Existing relationships between respondents and VCs laid the groundwork of trust for respondents to share their HIVST results directly with VCs and discuss the next steps. Respondents felt comfortable sharing their HIVST results directly with their VCs, who for many clients, uploaded it to the virtual platform. They also discussed their results, reactions and next steps with VCs (Table 2).

#### 3.4.7 Screening positive

Respondents who screened positive accepted emotional support and guidance from their VC as they struggled with feelings of shock and worry after testing positive. VCs helped navigate confirmatory testing and treatment initiation. Respondents discussed challenges with linking to care following a positive screening result. Challenges included long lines and waiting times to receive test results at government confirmatory testing sites, being scared of a positive confirmatory test and fearing judgemental or invasive questions from providers. Respondents who were confirmed to be living with HIV discussed reasons for delays in starting treatment, such as feeling scared, not being mentally or emotionally ready, or worrying that their family will find out they are living with HIV. Resistance to starting treatment which respondents shared in interviews stemmed from fear of invasive questions from public providers, feeling emotionally/mentally unprepared, and feeling healthy and fine without treatment (Table 2).

## 4 DISCUSSION

Findings from our web‐based HIVST programme in India add to global evidence demonstrating the feasibility and acceptability of HIVST among key populations [13, 14]. Our programme shows the potential of web‐based channels with VC support to reach clients with HIVST (especially younger clients), support self‐efficacy to complete HIV screening, capture results and enable appropriate service linkage. Qualitative evaluation findings are most representative of web‐based client experiences and converge to demonstrate overall satisfaction with the programme and added value of VCs. The flexible and personalized support provided to HIVST clients through VCs in our programme addresses previously documented concerns among self‐testers about the inherent lack of counselling and potential for user error [13, 15] and may improve linkage to care and treatment for those who test positive.

Our programme builds on previous HIVST programmes offering ways to reach clients. The pilot Self‐Testing AfRica (STAR) initiative, initiated in 2015 in Malawi, Zambia and Zimbabwe, documented challenges to track positivity and service linkage among clients using data from HIVST distributors and clinics [16, 17]. In contrast, our programme tracked outcomes from clients directly. The high proportion of HIVST clients with documented results provided a more accurate estimate of HIV positivity. Other HIVST distribution initiatives have yielded HIV positivity similar to standard testing among comparable population groups [18]. However, our HIVST platform revealed a 6% screening positivity among clients, which is about 27 times higher than the general population prevalence in India and about two times higher than the national estimated HIV prevalence among men who have sex with men [19]. Studies in New Zealand and the United States similarly found higher HIV prevalence among men who have sex with men through online HIVST ordering and mail distribution compared to traditional HIV testing modalities [20, 21]. This supports the role of web‐based channels combined with personalized support from a VC to improve access to HIV screening and optimize linkage to prevention and treatment services among difficult‐to‐reach populations.

Virtual approaches are promising to reach key populations; however, programmes must keep in mind the hetero‐cis‐normativity pervasive in India and resulting minority stress for men who have sex with men and transgender communities [22, 23, 24, 25]. This adds to the widespread HIV‐related stigma and discrimination present in India making HIVST services highly sensitive. This played out for clients of our platform, many of whom were recruited from gay dating apps, who were particularly concerned about aspects of privacy. Our programme aimed to overcome these challenges by respecting the communication preferences of clients, delivering HIVST kits to any address clients chose, wrapping kits in discreet packaging and ensuring clients were contacted with the same VC throughout the process to support building trust. Our programme's experience adds to global evidence demonstrating the need for public health services to promote privacy for sexual and gender minority groups [18, 26].

Privacy is particularly important to consider for web‐based health programmes. While web‐based self‐testing platforms bring the potential of increased autonomy for proactive health maintenance, digital platforms introduce important considerations regarding the use of and access to health information [27]. Our findings add to existing evidence that clients of digital self‐testing programmes have concerns about privacy with their health data [28, 29]. Public health services and interventions should give comprehensive attention to data privacy and data security in virtual strategies. Services should be open to clients as much as possible about what personal information is gathered when engaging with online interventions and how it will be used, offering clients the chance to opt‐out.

The role of VCs in our programme proved critical to help clients navigate concerns about privacy, as well as support their overall experience with HIVST. Initial outreach by someone who a client is likely to trust, such as a community member or peer, can support subsequent engagement. Community‐oriented counsellors answered clients’ questions about HIVST and the logistics of the virtual platform. They helped clients feel comfortable while doing HIVST and handle challenging emotions such as fear of judgement and anxiety surrounding a positive HIV test result. Studies in other global settings similarly highlight the critical role of personalized support from a trusted peer or trusted counsellor to support HIV testing [30, 31, 32, 33]. Building on these learnings, our programme demonstrates the importance of building rapport and trust between counsellors and clients which can enhance client experiences and support more clients to report HIVST results through virtual platforms.

HIVST can uniquely fill gaps of access for key population groups, but this may look different for each individual. The COVID‐19 pandemic only aggravated systemic disadvantages faced by sexual and gender minorities in India, especially access to preventative services like HIV testing [34]. At the time of programme implementation, HIVST was positioned to fill a gap of service access in new ways than before the pandemic [35]. Technology helped clients access HIV screening while avoiding travel and in‐person interactions. However, respondents in interviews shared concerns that this approach excludes people in their communities with limited literacy and smartphone access. This highlights the need for HIVST programmes to accommodate varying levels of technology access and literacy. Our programme considered this by offering virtual support and offering in‐person HIVST access at PUPs, which helped diversify levels of access and languages that services could accommodate. Trained counsellors at PUPs supported clients to register on the digital platform and complete HIVST, if they desired to test at the PUP. Similarly to VCs, counsellors at PUP locations helped clients navigate concerns, questions and offered emotional support. PUP staff served a key role to expand access to HIVST for clients who may not be as comfortable using web‐based platforms as others. This may be especially important given the higher positivity we saw among clients who accessed HIVST from in‐person locations.

Our programme learned ways we can further support clients’ needs and build trust. These included expanding local languages available on the platform's website and on kit instructions. Our programme offered materials in Hindi, English and Telugu but given the diverse languages spoken across India, more language options are needed. Further, having a trusted health expert endorse the accuracy of HIVST would likely increase client trust and the perceived efficacy of HIVST as a dependable technology. Previous HIVST acceptability studies in India similarly demonstrate the need to integrate counselling as well as address language diversity and kit accuracy concerns [36, 37, 38]. To support varied needs across target communities, HIVST programmes should adopt a differential service delivery approach [39], offering diverse modalities of communication and service delivery tailored to each person and their context, and continue to explore ongoing barriers to service linkage.

### 4.1 Limitations

Limitations of our programme include most clients being men who have sex with men. This is a result of the outreach and demand generation conducted by VCs on gay dating applications popular in India. Outreach to other populations is a focus of future implementation. The majority of the assessment participants received their kit via courier, which reflects our recruitment mostly through online channels rather than PUPs. Our assessment findings may be limited to those who are more likely to use web‐based channels than in‐person services for HIVST and who were open to sharing their experiences. Further, our assessment findings reflect the experiences of those who consented to offer feedback and completed an interview. Out of the 244 clients we reached to ask about completing an interview, 41 clients consented to an interview (17%). Challenges to recruit interview clients included clients not being willing to talk, not being interested in completing an interview and not having time. People may have declined to complete an interview because the format of an in‐depth conversation was different than past experiences with the online service platform. People may have chosen to participate in our virtual service platform for the same reasons they declined an interview, such as not wanting interactions with health staff or not wanting to answer questions about themselves. This further highlights the opportunity of online service platforms to reach people who value discretion and fewer interactions with providers. Learnings are likely more representative of people who had positive experiences with our services and may be more inclined to access health services, compared to people with negative experiences or negative perceptions of healthcare. The qualitative interviews were conducted only in Hindi or English. This restricted who we were able to include in the assessment to those who spoke these languages, limiting   the linguistic representation of evaluation findings. Given the challenges to recruit clients who screened positive after HIVST to participate in our evaluation, more work is needed to explore how to best support linkage from screening to confirmatory testing to treatment services. Further work is also needed to understand values and preferences surrounding HIVST services and online platforms among people who prefer less interaction with health services and staff.

## 5 CONCLUSIONS

Our programme experience highlights the role of web‐based HIVST services with personalized VC support to reach key populations with a higher HIV burden. With increasing online engagement and uptake of telemedicine globally, self‐testing services offer a critical approach to achieve targets for infectious disease programmes across diagnosis and linkage to care and treatment.

## COMPETING INTERESTS

SSS received research grants and products for his institution from Gilead Sciences and research grants and products from Abbott Laboratories outside of the submitted work. SSS received consulting fees from Gilead Sciences outside of the submitted work. All other authors have no competing interests to declare.

## AUTHORS CONTRIBUTION

JT, RPK, JB, SHM, AKR, MRP, SCG, AS, SSS and AMM conceptualized and designed the programme and evaluation. SK and DSJ provided inputs to the design and execution of the programme and assisted with partnering with other agencies. JT, JB, TAL, RPK, SO, UG, SHM, AKR, VA, ST, MRP, SCG, AS, SSS and AMM collected data and conducted data analysis. SSS acquired the funding. JT, JB, RPK, SO, SHM, AKR and AMM accessed and verified all study data. JT, RPK, JB, SSS and AMM wrote the original draft of the manuscript. All authors had access to data used in the manuscript, supported revision and editing of the manuscript, and accepted responsibility to submit for publication.

## FUNDING

The study was supported by the United States Agency for International Development India Cooperative Agreement Number 72038619CA00001.

## Data Availability

The data presented in this paper are not publicly available. Individual participant data that underlie the results reported in this article, after de‐identification, are available from the corresponding author upon reasonable request.
